# Immunohistochemical Changes in MMR Status, ER/PR, and p53 Expression in Recurrent Endometrial Carcinomas

**DOI:** 10.3390/medicina62050828

**Published:** 2026-04-27

**Authors:** Zeliha Guzeloz, Ozce Kutlu, Ozgur Erdogan, Gonul Demir, Bugra Taygun Gulle, Muzaffer Sancı, Mihriban Erdogan, Canan Kelten Talu

**Affiliations:** 1 Department of Radiation Oncology, İzmir City Hospital, Izmir 35540, Türkiye; gonuldemir1990@gmail.com; 2 Department of Pathology, Tepecik Training and Research Hospital, Izmir 35020, Türkiye; ozcekutlu@gmail.com; 3 Department of Gynecologic Oncology, İzmir City Hospital, Izmir 35540, Türkiye; ozgrerd@gmail.com; 4 Public Health Department, Provincial Health Directorate, Izmir 35250, Türkiye; gullebugrataygun@gmail.com; 5 Department of Gynecologic Oncology, Izmir Faculty of Medicine, University of Health Science Türkiye, Izmir 35100, Türkiye; drsanci@yahoo.com; 6 Department of Radiation Oncology, Izmir Faculty of Medicine, University of Health Science Türkiye, Izmir 35100, Türkiye; mihribanerdogan73@gmail.com; 7 Department of Pathology, Izmir Faculty of Medicine, University of Health Science Türkiye, Izmir 35100, Türkiye; canankelten@gmail.com

**Keywords:** endometrial cancer, mismatch repair, ER/PR, p53, recurrence

## Abstract

*Background and Objectives:* The aim of this study is to assess the mismatch repair (MMR) status and immunohistochemical changes in cases of recurrent endometrial cancer following primary surgery and to evaluate the impact of these changes on prognosis. *Materials and Methods:* Thirty-one patients diagnosed with endometrial cancer who underwent surgery and had pathologically confirmed recurrences were evaluated. Changes in MMR protein expression, estrogen receptor (ER)/progesterone receptor (PR), and p53 expression in primary surgery and recurrent tumor tissues were assessed using immunohistochemical methods. Prognostic factors influencing these parameters and survival data were investigated. *Results*: In the assessment of recurrent materials, there were four cases where the MLH-1&PMS-2 staining status changed from intact to loss and four cases that changed from loss to intact. No changes were observed in regard to MSH-2 &MSH-6 staining. The ratios of pMMR and dMMR following the primary surgery were 55% (n = 17) and 45% (n = 14), respectively. Four cases transitioned from pMMR to dMMR, and four cases transitioned from dMMR to pMMR. After recurrence, changes in the ER, PR, and P53 status were observed in seven, three, and three patients, respectively. *Conclusions*: Changes in the MMR status, receptors, and P53 were observed. It is necessary to re-evaluate prognostic parameters via biopsies in recurring cases and to adjust rescue treatments accordingly.

## 1. Introduction

The primary treatment for endometrial cancer is surgery. Although adjuvant treatment options are determined based on prognostic factors, such as age, stage, lymphovascular invasion, grade, myometrial invasion, and tumor size, their efficacy remains a subject of debate [1,2]. In prognoses, the presence of heterogeneity, even within the same stage, highlights the need for new prognostic markers in endometrial cancer [3].

The Cancer Genome Atlas Research Group’s genomic characterization of endometrial cancer marks the first step in this direction. Their study identified four molecular subgroups: a group with POLE mutations, demonstrating the best prognosis; a hypermutated/microsatellite instability group; a low copy number group, exhibiting type 1 endometrioid-like behavior; and a high copy number/p53 mutant group, exhibiting serous carcinoma-like behavior with the poorest prognosis [4].

Microsatellite sequences are defined as sequences consisting of 1–6 base pairs in the DNA sequence [5]. Errors occurring in this region are corrected by the mismatch repair system (MMR). The primary MMR proteins are MLH-1 and MSH2, and their regulators are PMS-2 and MSH-6 [6]. A synthesis malfunction in any of these four proteins is termed an MMR error. A loss of staining of MMR proteins is referred to as deficient MMR (dMMR), while the preservation of nuclear staining is termed proficient MMR (pMMR) [7].

For recurrent endometrial cancer, options for salvage treatment include surgery, radiotherapy, chemotherapy, hormone therapy, immunotherapy, and targeted therapy, though they are not standardized. Decisions are often made based on the characteristic or type of tumor tissue retrieved during the primary excision, often depending on tissue availability and/or attempts to avoid an additional biopsy of the recurrent site [8]. However, the re-classification of molecular profiles and immunohistochemical evaluations in patients with recurrent disease is critical to plan advanced treatments. The American Society for Radiation Oncology (ASTRO) guidelines provide oncological treatment recommendations according to the new immunohistochemical classification. The guidelines state that patients with dMMR tumors do not benefit from chemotherapy but may benefit from external radiotherapy, emphasizing that dMMR tumors are immunogenic, particularly in metastatic diseases where immunotherapies like pembrolizumab are highlighted [9,10]. It is noted that p53 mutant tumors benefit from chemotherapy [9].

There is limited research on the pathological changes in recurrent endometrial cancer tissues. These studies have found that microsatellite instability is greater in recurrent cases compared to primary ones, with initially stable cases becoming unstable upon recurrence and differences in the MMR status between primary and recurrent cases [8,11,12].

The aim of this study is to determine the mismatch repair status and immunohistochemical changes in the paired tissue of primary and recurrent endometrial cancer from the same patients and assess the impact of these changes on their prognosis.

## 2. Patients and Methods

### 2.1. Patients

At our institution, all patients diagnosed with endometrial cancer and undergoing staging surgery are evaluated by a multidisciplinary council for adjuvant treatment decisions. In our ESGO-accredited training center during the period in which this study was conducted, post-operative follow-ups are recommended every 3 months for the first two years, every 6 months for years 2–5, and annually thereafter. These follow-ups always include a gynecological examination, an annual Pap smear, and radiological assessments as per the stage of the disease. In patients with suspected recurrence based on gynecological examination or radiological findings, excisable lesions are removed, and for those not suitable for excision (medically inoperable, previously received radiotherapy making excision inappropriate, or unable to be excised due to anatomical localization), further treatments are planned without excisions if the cases are radiologically verified as recurrence.

### 2.2. Methods

In our study, we reviewed the oncology council records of approximately 3000 patients diagnosed with endometrial cancer who underwent surgery (via laparotomy) at our center between 2009 and 2023. A total of 31 patients with pathologically confirmed (biopsy or excision) recurrences were included in this study. Ethical approval was obtained from the Tepecik Training and Research Hospital Ethics Committee (approval number: 2023/07-02). Patients 18 and older who were diagnosed with endometrial cancer and who underwent primary surgery and had accessible excision/surgical pathology specimens following recurrence were included. Patients with de novo stage 4 cancer and those whose primary or recurrent pathology specimens were inaccessible or could not be technically evaluated were excluded.

#### 2.2.1. Tissue Samples

Tissue samples were obtained retrospectively by the same expert pathologist from the archive of the pathology department after both primary surgery and recurrence. These tissues were evaluated for the expression status of mismatch repair proteins (MMR: MLH1, PMS2, MSH2, and MSH6) as well as for estrogen receptors (ERs), progesterone receptors (PRs), and p53 using the immunohistochemical staining method. The following antibodies (with clones) were used: MLH1 (M1); PMS2 (A14-4); MSH2 (G219-1129); MSH6 (SP93); ER (SP1); PR (1E2); and p53 (BP-53-11).

**Figure 1:**
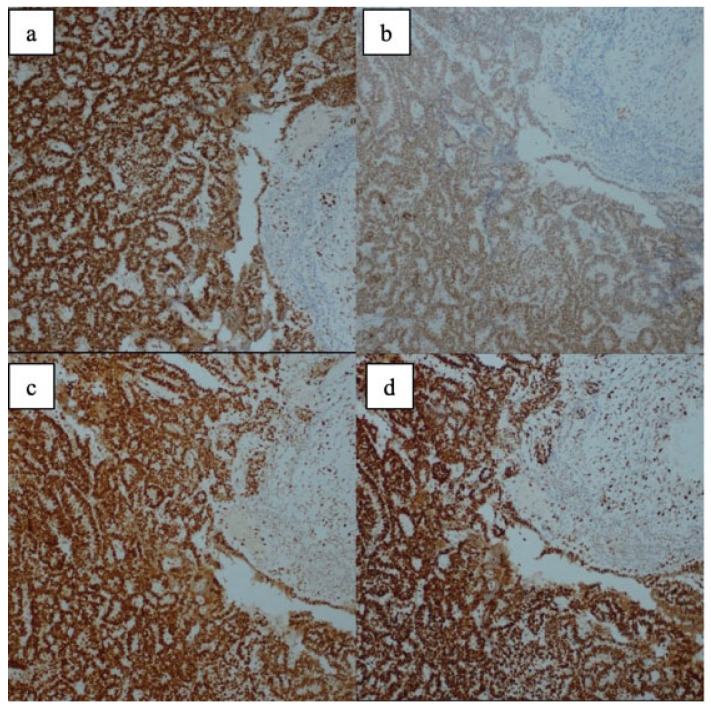
Case 1—Tumor cells exhibit nuclear positivity for MLH1 (**a**), PMS2 (**b**), MSH2 (**c**), and MSH6 (**d**). Mismatch repair proteins are intact (pMMR).

**Figure 2:**
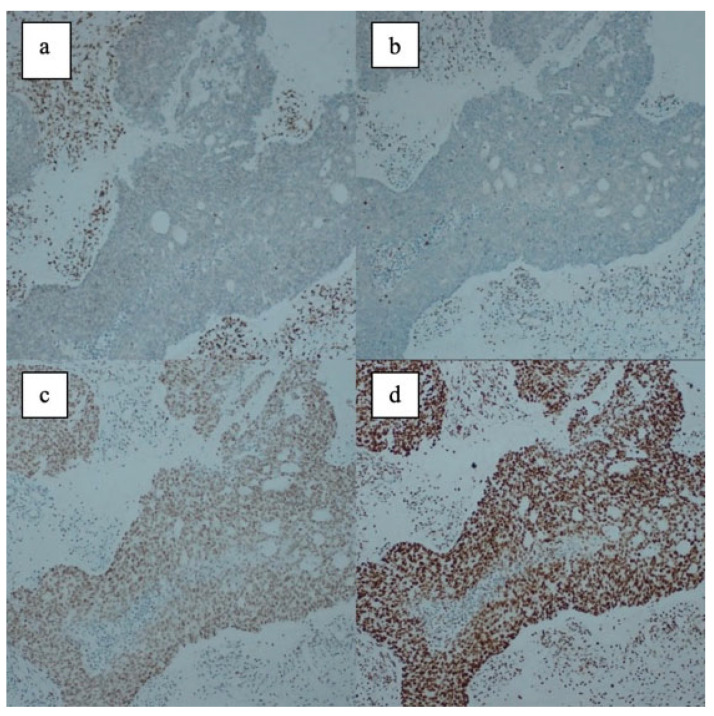
Case 2—There is no nuclear staining for MLH1 (**a**) and PMS2 (**b**) (internal control present), but there is staining for MSH2 (**c**) and MSH6 (**d**). Mismatch repair protein loss (dMMR).

MMR proteins were categorized as either intact/proficient MMR (pMMR) or negative/deficient MMR (dMMR). Intact expression for MMR proteins (pMMR) was defined as the presence of any amount of nuclear staining within tumor cells. Negative expression (dMMR) was defined as the complete absence of nuclear staining within all tumor cells with concurrent positive staining for internal control cells such as normal endometrial glandular/stromal cells or infiltrating lymphocytes. Examples of immunohistochemical MMR evaluation images can be found in the figures below: Figure 1 presents the pathological appearances of the immunohistochemical MMR tests for Case 1, while Figure 2 presents those for Case 2.

The ER/PR status was defined as the percentage of positively stained tumor cells within the entire invasive tumor area. According to the protocol, the threshold value is ≥%1. p53 was categorized as either mutant or wild-type, where mutant-type p53 staining was defined as an overexpression pattern, null pattern, or cytoplasmic staining [13]. Additionally, the status of MELF (microcystic elongated and fragmented), a pattern of myometrial infiltration that may mimic lymphovascular space invasion, was noted in H&E sections in primary excision materials.

#### 2.2.2. Clinical Data

Recorded data included age at diagnosis, stage (according to the International Federation of Gynecology and Obstetrics FIGO-2017 Staging System), grade, surgical details, adjuvant treatments, and recurrence sites. Overall survival was calculated from the date of the biopsy to the last follow-up.

#### 2.2.3. Statistics

Prognostic factors, adjuvant treatments, and survival parameters that could affect the outcome were analyzed. Statistical analysis was conducted using Statistical Package for the Social Sciences (SPSS) version 24.0 (International Business Machines Corporation [IBM], Chicago, IL, USA).

The Shapiro–Wilk test was used to assess normal distributions, and the Mann–Whitney U and chi-square tests were used for comparisons between two groups of non-parametric data. A Kaplan–Meier analysis was performed for survival analysis, and a *p*-value of <0.05 was considered statistically significant.

## 3. Results

The mean age in this study was 62.2 ± 9.8. All patients underwent total abdominal hysterectomy and bilateral salpingo-oophorectomy. Lymph node dissection was performed in all except four patients. Of those who did not undergo lymph node dissection, three patients were at stage IA and one was at stage IB. The patient at stage IB did not undergo lymph node dissection due to superficial invasion observed during a frozen section examination. The final pathology report identified lymph node positivity in three patients (9.6%). Table 1 displays the pathology and treatment characteristics post-primary surgery.

A total of 13 local (at the cuff level), 14 regional, and 3 distant recurrences were identified. Table 2 displays the immunohistochemical evaluation for each patient after primary surgery and recurrence.

After the primary surgery, the intact staining rates for MLH1 and PMS2 were 55% (n = 17); the rates for MSH2 and MSH6 were 100% (n = 31). In the evaluations of the recurrence tissues, there were four cases in which the MLH1 and PMS-2 staining changed from intact to loss and four cases where it changed from loss to intact. No changes were observed for the MSH-2 and MSH6 staining. These details are reflected in Table 3.

In our study, changes in the MMR status were observed in 8 patients, whereas no changes were detected in 23 patients. Among these 23 patients, 13 were initially pMMR before primary surgery and remained pMMR at recurrence (consistent pMMR), while 10 were initially dMMR before primary surgery and remained dMMR at recurrence (consistent dMMR).

After primary surgery, the rates of proficient MMR (pMMR) and deficient MMR (dMMR) were 59% (n = 17) and 39% (n = 14), respectively. There were four cases of discordance from pMMR to dMMR and four cases from dMMR to pMMR (*p* = 0.687). The changes in MMR status are detailed in Table 4.

After the primary surgery, 97% of patients were ER-positive, 87% were PR-positive, and 93.5% had wild-type p53. Following recurrence, changes in ER were observed in seven cases (six cases from positive to negative; one case from negative to positive), changes in PR were observed in three cases (one case from negative to positive), and changes in P53 status were observed in three patients (three cases from wild to mutant), with no statistical significance observed (*p* = 0.642, *p* = 0.550, *p* = 1) (Table 5). The MELF pattern was not determined in any cases following primary surgery.

The ER/PR status was classified as either positive or negative, while the p53 status was categorized as either wild-type or mutant.

Only the median age was found to be a statistically significant factor affecting MMR status changes (*p* = 0.019). We observed a higher median age in the group with MMR status changes (median age 70.5 vs. 61).

In cases without MMR changes, the wild-type p53 status remained the same. However, in cases with MMR changes, the p53 was found to be mutant in two cases (33.3%), both of which were diagnosed as mixed carcinoma.

No statistically significant difference was observed in the MMR change status between cases with distant recurrence (liver, breast, and pleura) and those with intra-abdominal recurrence (*p* = 1.000). However, it was noted that two out of the three cases of distant recurrence had deficient MMR (dMMR) present in both primary and recurrent tumor samples.

**Figure 3:**
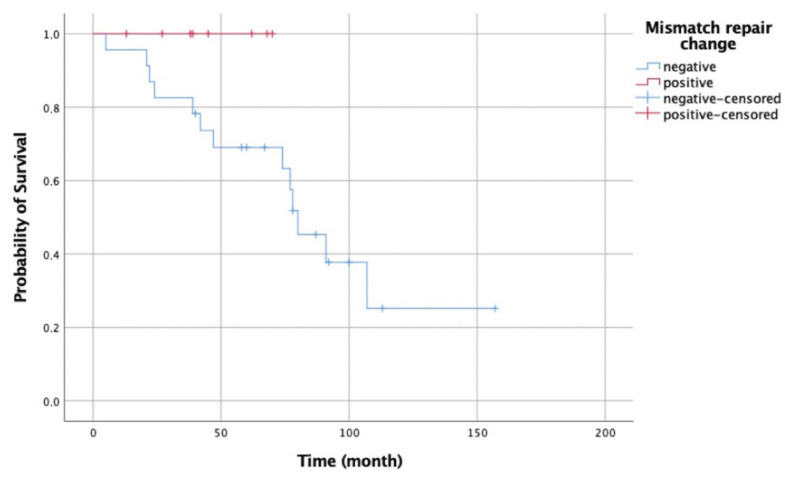
Survival graph based on MMR change status.

The median survival time for the total 31 patients was 80 months (95% CI: 60.2–99.8). No deaths were observed in the 8 cases with MMR changes, while 13 deaths were observed among the 23 cases without MMR changes. No statistically significant difference in survival was detected between the groups (*p* = 0.154) (Figure 3).

**Figure 4:**
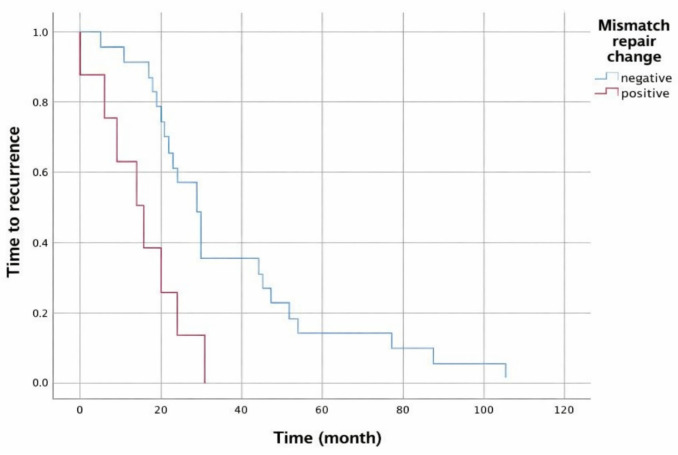
Time to recurrence graph based on MMR change status.

When considering the time between the primary surgery and recurrence as a distribution plot of the time to recurrence, a comparison between groups with and without MMR changes shows that the median time to recurrence in the group with MMR changes was 14 months (95% CI: 4.3–23.7), while in the group without MMR changes, it was 29 months (95% CI: 23.3–34.6) (*p* = 0.003) (Figure 4).

## 4. Discussion

In our study, we evaluated the changes in the MMR proteins (MLH1, PMS2, MSH2, and MSH6), ER/PR levels, p53 status, and MELF patterns from tissue samples collected after primary surgery and at recurrence in patients with local and regional recurrences. While the median age in the group with MMR changes was statistically significant, the small sample size limited statistical significance in other parameters. However, changes in the immunohistochemical and receptor levels mentioned above were observed. Moreover, it was found that the time to recurrence was statistically longer in the group with no change in their MMR status. In subgroup evaluations, we determined that two cases with mutant p53 in the non-MMR group were mixed carcinomas, and among three cases with metastasis, two were found to be dMMR post primary surgery and at recurrence.

The literature on MMR and microsatellite instability following primary surgery and recurrence is limited. Moroney et al. examined the microsatellite instability and MMR status in cases of recurring endometrial cancer compared to a control group and identified high microsatellite instability in recurring cases [11]. Stasenko et al. reported on a patient with a POLE mutation who was initially microsatellite stable; the patient developed metastasis during follow-up, and a subsequent examination revealed a conversion to high microsatellite instability, leading to an adjustment in treatment based on these new findings [12]. In a series of 34 patients with endometrioid, serous, and mixed carcinomas, Soslow et al. detected a loss in MMR evaluations [14]. Pijenburg et al. did not observe any differences in microsatellite instability or MLH1 and MSH2 expression [15].

In a study by Ta et al. [8], a 7% discordance rate was identified between primary and recurrent/metastatic tumors regarding the mismatch repair status. Our study found a higher discordance rate of 12.9% in the group transitioning from pMMR to dMMR.

Our study also revealed that the two patients undergoing MMR changes (proficient to deficient) were classified as mutant p53 post-primary surgery, and both were diagnosed with mixed carcinomas (endometrioid and serous tumors). We believe that for mixed histology endometrial cancer with p53 positivity, even if the MMR status is preserved at the initial surgery, the MMR status should be re-evaluated at recurrence, and treatment should be planned by considering these aggressive pathological findings.

In the three cases with detected distant metastases, a loss of MMR staining was noted between the primary surgery and recurrence. We believe that this change is associated with distant metastasis, indicating a poor prognosis. Cases with a detected MMR deficiency at recurrence should be closely monitored for distant disease, and the need for systemic treatment should be evaluated.

In our study, we observed that the group with changes in their MMR status had a shorter time to recurrence (14 months vs. 29 months) compared to the group without changes. This suggests that changes in the MMR status during recurrence, reflecting alterations in cellular biology and subclonal expression, lead to more aggressive tumor behavior and a shortened time to recurrence. In our study, although time to recurrence was shorter in the group with MMR changes, there were no deaths in this group. In contrast, deaths were observed in the group without MMR changes. We hypothesize that this situation, which cannot be explained by the patients’ treatment and clinical characteristics, might be due to the fact that groups without MMR changes were not POLE mutant cases. Therefore, groups without MMR changes may have a worse prognosis, leading to the observed deaths.

It is believed that tumors with deficient MMR (dMMR) are more responsive to radiotherapy and immunotherapy [16,17]. Therefore, assessing the MMR protein status in recurring tumors is crucial for planning salvage therapies [10].

Although no significant changes were observed in the ER/PR status after recurrence, the fact that the majority of recurrent cases were positive for both receptors suggests a consideration of hormone therapy. The impact of PR positivity on overall survival remains unclear; however, progestin replacement is a treatment option for non-recurring endometrial cancer [18]. It is also suggested that hormone therapy may be viable for ER-positive cases [19]. The results of the RAINBO study are anticipated to further clarify the role of hormone therapy in endometrial cancer [20].

Our study’s retrospective design introduces some limitations. The primary limitation is the small number of patients. Complications and the difficulty of performing a second surgery in patients who have already undergone surgery and received radiotherapy previously make it challenging to differentiate between cases of pathologically and radiologically detected recurrences. As our institution is a referral hospital, we accept many cases from external centers. The inability to obtain slides and paraffin blocks from the institutions where the primary surgery was performed limited the number of patients to 31. The lack of uniformity in the histological subtypes of the evaluated specimens is seen as a disadvantage for homogenization. However, our main aim was to identify changing patterns from post-primary surgery to recurrence; hence, all cases except those with de novo stage 4 disease were evaluated. Moreover, immunohistochemical staining was conducted for four mismatch repair proteins (MLH1, PMS2, MSH2, and MSH6) in tumor tissues from excision materials of primary and metastatic foci, ensuring internal or external control. However, cases with differing MMR protein expressions were not further verified via PCRs in relation to the microsatellite instability status. Additionally, MLH1 methylation testing was not performed for cases exhibiting a concurrent loss of MLH1 and PMS2 expression.

Another limitation is the lack of evaluation for POLE mutations, due to costs and the lack of reimbursement within the national health financing system, preventing us from incorporating this as a routine part of our evaluation. Given that our study group consisted of recurrent cases, which typically have poor prognoses, based on the literature we assumed that they would likely not be classified as POLE mutants.

Despite these limitations, our study is among a limited number in the literature that concurrently explore the status of MMR mismatch repair proteins, receptor profiles, p53, and MELF patterns in primary surgery and recurrence pathology materials. Despite the small number of cases, a comprehensive evaluation of all cases was achieved.

## 5. Conclusions

Considering that the immunohistochemical characteristics of the tumor can change after recurrence, we believe it is crucial that patients who are able to undergo tissue diagnosis have their recurrence pathologically verified. This allows for a review of prognostic parameters and an adjustment of salvage treatments accordingly. Given the rapid expansion of treatment options, including radiotherapy, systemic therapies, targeted agents, and immunotherapies, each new piece of data obtained from patients with recurrence can potentially open doors to alternative treatments.

## Figures and Tables

**Table 1:** Pathological data and treatment characteristics.

Parameters	% (n)
**Pathology**	
Endometrioid	90.3% (n = 28)
Mix (endometrioid + serous)	6.5% (n = 2)
Carcinosarcoma	3.2% (n = 1)
**Stage**	
I	80.6% (n = 25)
II	3.3% (n = 1)
III	16.1% (n = 5)
**Grade**	
1	19.4% (n = 6)
2	54.8% (n = 17)
3	25.8% (n = 8)
**Deep Invasion**	
Present	54.8% (n = 17)
Absent	45.2% (n = 14)
**Lymphovascular Invasion**	
Present	55% (n = 17)
Absent	42% (n = 13)
Unknown	3% (n = 1)
**Adjuvant Radiotherapy**	
External RT and brachytherapy	32.5% (n = 10)
External RT	19% (n = 6)
Brachytherapy	16% (n = 5)
No radiotherapy	32.5% (n = 10)
**Salvage Pelvic RT**	16% (n = 5)
**Adjuvant Chemotherapy**	
Administered	29% (n = 9)
Not administered	71% (n = 22)

**Table 2:** Primary surgery and recurrence assessment of MMR proteins.

	Primary Surgery			Recurrence			
Case	MLH-1 & PMS-2	MSH-2 & MSH-6	MMR Status	MLH-1 & PMS-2	MSH-2 & MSH-6	MMR Status	Change in MMR Status
1	+	+	pMMR	+	+	pMMR	no
2	−	+	dMMR	−	+	dMMR	no
3	+	+	pMMR	+	+	pMMR	no
4	+	+	pMMR	+	+	pMMR	no
5	−	+	dMMR	−	+	dMMR	no
6	+	+	pMMR	+	+	pMMR	no
7	−	+	dMMR	−	+	dMMR	no
8	+	+	pMMR	+	+	pMMR	no
9	−	+	dMMR	−	+	dMMR	no
10	−	+	dMMR	−	+	dMMR	no
11	+	+	pMMR	+	+	pMMR	no
12	+	+	pMMR	+	+	pMMR	no
13	−	+	dMMR	−	+	dMMR	no
14	−	+	dMMR	+	+	pMMR	yes
15	−	+	dMMR	−	+	dMMR	no
16	−	+	dMMR	−	+	dMMR	no
17	+	+	pMMR	−	+	dMMR	yes
18	+	+	pMMR	−	+	dMMR	yes
19	+	+	pMMR	+	+	pMMR	no
20	+	+	pMMR	+	+	pMMR	no
21	+	+	pMMR	+	+	pMMR	no
22	−	+	dMMR	+	+	pMMR	yes
23	+	+	pMMR	+	+	pMMR	no
24	−	+	dMMR	−	+	dMMR	no
25	+	+	pMMR	−	+	dMMR	yes
26	−	+	dMMR	−	+	dMMR	no
27	+	+	pMMR	+	+	pMMR	no
28	−	+	dMMR	+	+	pMMR	yes
29	+	+	pMMR	−	+	dMMR	yes
30	−	+	dMMR	+	+	pMMR	yes
31	+	+	pMMR	+	+	pMMR	no

+ intact; − loss.

**Table 3:** Changes in immunohistochemistry.

	Primary Surgery (n)		Recurrence (n)		Changes (n)
	Intact	Loss	Intact	Loss	Intact to Loss Loss to Intact
**MLH** **1/PMS2**	17	14	17	14	4 4
**MSH2/MSH6**	31	0	31	0	0 0

**Table 4:** MMR change table.

	Primary Surgery (n)	Recurrence (n)	Changes pMMR to dMMR dMMR to pMMR	No Changes
**p** **MMR**	17	17	4	13
**dMMR**	14	14	4	10
**Total**	31	31	8	23

**Table 5:** ER/PR and p53 status.

	Primary Surgery (n)		Recurrence (n)			
	Positive	Negative	Positive	Negative	Changes	*p*
**ER**	30	1	25	6	7	0.642
**PR**	27	4	24	7	3	0.550
	**Wild**	**Mutant**	**Wild**	**Mutant**	**Changes**	
**p53**	29	2	28	3	3	1

## Data Availability

The original contributions presented in this study are included in the article. Further inquiries can be directed to the corresponding author.
